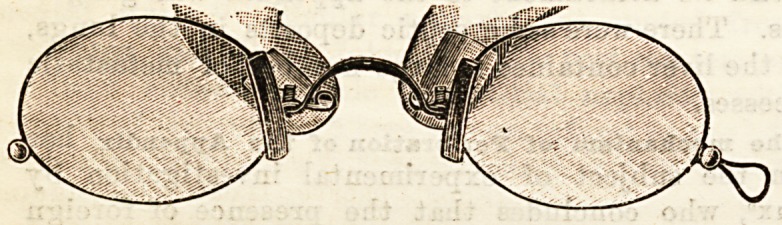# New Appliances and Things Medical

**Published:** 1895-05-11

**Authors:** 


### NEW APPLIANCES AND THINGS MEDICAL.

[We shall be glad to receive, at our Office, 428, Strand, London, W.C., from the manufacturers, specimens of all new preparations and appliances which may be brought out from time to time.]

### MOVILLETTE EYE-GLASS.

We have received for criticism "the new Movillette eye-glass" from Messrs. J. Raphael and Co., manufacturing opticians, of 13, Oxford Street. It is intended to secure immovable foci in pince-nez form, and to be better adapted to any shaped nose than the ordinary eye-glass. The accompanying illustration shows how this is accomplished. The bridge between the lenses is rigid, and the glasses are fixed upon the nose by means of two plaquets, regulated by two springs, which are pressed together by finger and thumb for adjustment. The idea is a good one, and probably many people who do not care to wear spectacles will find the "Movillette" a convenient substitute on account of the horizontal position. We have tried them and find the pressure of the nose pieces rather too dragging for perfect comfort, though we should think this might be obviated by a lighter tension of the springs. Another objection is the projection of the "spurs" in front, which come slightly into the range of vision; this is, however, a detail which could probably be modified. The eye-glasses are very neat in appearance.

### VICHY WATERS.

(MESSRS. INGRAM AND ROYLE, 52, FARRINGDON STREET.)

We are informed that, acting on the suggestion of many customers, the Cie de Vichy have reduced the trade price of the half-bottles of the Vichy Waters, this size being so much more convenient to consumers, as in many instances a large bottle is opened when the smaller would suffice. Considering that the small bottles are half-litres (not "reputed pints") the price of Vichy Water is now about the same as the "table waters."

## Figures and Tables

**Figure f1:**